# Differences in renal cortex transcriptional profiling of wild-type and novel type B cystinuria model rats

**DOI:** 10.1007/s00240-022-01321-6

**Published:** 2022-04-13

**Authors:** Zihan Zhang, Rui Zheng, Zhoutong Chen, Xia Zhan, Xiaoliang Fang, Meizhen Liu, Yongmei Li, Yonghu Xu, Dali Li, Hongquan Geng, Xiaohui Zhang, Guofeng Xu

**Affiliations:** 1 Department of Pediatric Urology, Xinhua Hospital Affiliated To Shanghai Jiao Tong University School of Medicine, 1665 KongJiang Road, Shanghai, 200092 China; 2 Department of Pediatric Endocrinology and Genetic Metabolism, Shanghai Institute for Pediatric Research, Xinhua Hospital, Shanghai Jiao Tong University School of Medicine, Shanghai, China; 3 Shanghai Key Laboratory of Regulatory Biology, Institute of Biomedical Sciences and School of Life Sciences, East China Normal University, Shanghai, 200241 China; 4 Fudan University Shanghai Cancer Center, Shanghai, China

**Keywords:** Cystinuria, SLC7A9, CRISPR-Cas9 system, Renal cortex, Transcriptional profiling

## Abstract

Cystinuria is a genetic disorder of cystine transport that accounts for 1–2% of all cases of renal lithiasis. It is characterized by hyperexcretion of cystine in urine and recurrent cystine lithiasis. Defective transport of cystine into epithelial cells of renal tubules occurs because of mutations of the transport heterodimer, including protein b^0,+^AT (encoded by SLC7A9) and rBAT (encoded by SLC3A1) linked through a covalent disulfide bond. Study generated a novel type B cystinuria rat model by artificially deleting 7 bp of *Slc7a9* gene exon 3 using the CRISPR-Cas9 system, and those *Slc7a9*-deficient rats were proved to be similar with cystinuria in terms of genome, transcriptome, translation, and biologic phenotypes with no off-target editing. Subsequent comparisons of renal histopathology indicated model rats gained typical secondary changes as medullary fibrosis with no stone formation. A total of 689 DEGs (383 upregulated and 306 downregulated) were differentially expressed in the renal cortex of cystinuria rats. In accordance with the functional annotation of DEGs, the potential role of glutathione metabolism processes in the kidney of cystinuria rat model was proposed, and KEGG analysis results showed that knock-out of *Slc7a9* gene triggered more biological changes which has not been studied. In short, for the first time, a rat model and its transcriptional database that mimics the pathogenesis and clinical consequences of human type B cystinuria were generated.

**Supplementary Information:**

The online version contains supplementary material available at 10.1007/s00240-022-01321-6.

## Introduction

Cystinuria is one of the most common inborn defects of metabolism in humans [1, 2]; it is predominantly inherited as an autosomal recessive trait. Cystinuria occurs in 1 per 7000 worldwide, taking 1% of all urolithiasis cases and 4–5% of urolithiasis cases in children [3]. Urine cystine concentrations of patients are elevated with defective reabsorption of dibasic amino acids (lysine, arginine, and ornithine) and especially of cystine through the brush-border membrane of the renal tubules [4]. Hyperexcretion of poorly soluble cystine in urine increased the risk of the development of cystine precipitation and stone in the urinary tract.

The cystine transport heterodimer consists of protein b^0,+^AT (encoded by gene SLC7A9) and rBAT (encoded by gene SLC3A1) that are connected by a covalent disulfide bond and are located in the brush-border membrane of the proximal renal tubule and intestine. Cystinuria is caused by mutations in b^0,+^AT or rBAT [5–7], classified into types A, B, and AB by the position of mutations. Mutations are type A if homozygous mutations occur in SLC3A1 and they are type B if homozygous mutations occur in SLC7A9. Putative type AB mutations are defined as single mutations found in each gene [8]. SLC3A1 heterozygotes showed no apparent phenotype, while urinary amino acid levels of SLC7A9 heterozygotes varied [9, 10]. Type B cystinuria is the most common type (53%) presenting an inheritance pattern of autosomal dominant [11].

Not all type B cystinuria patients present with renal stones; however, they might develop stones in the context of low urine volumes or high intake of animal protein [2]. Up to 70% of patients with cystinuria develop chronic kidney disease (CKD), culminating in end-stage renal disease [12, 13]. Medical treatments of cystinuria aim to lower the absolute amount of cystine and increase its solubility [9, 10, 14]; unfortunately, all these treatments produce unfavorable outcomes, and gene therapy research, which might turn out to be a fundamental and practicable approach, is currently in the early stages.

Animal models can provide pathways for evaluating the safety and efficacy of new therapies. To date, except for several natural models (cat, dog and wolf, all of which are suboptimal for human studies) [15–17], several mouse models of cystinuria, including two kinds of *Slc3a1*-deficient mice (exon 1 deletion [18] and D140G induced mutation [19]), type B *Slc7a9* knockout mice (exons 3–9 deletion [20]) and type AB cystinuria heterozygous mice [21] (*Slc3a1*^±^, *Slc7a9*^±^). Previous studies have elucidated the molecular basis of cystinuria; however, studies of genotype–phenotype correlations require information from different animal models of each genotype.

Therefore, in the present study, to obtain more liquid samples and to facilitate observation and operation, we used rat models, which are more appropriate for metabolism and pharmacological studies. We reported the generation of the first rat model for type B cystinuria using the CRISPR-Cas9 system. Based on our rat model characterized by extremely high urinary cystine levels, we profiled the transcription alterations of cystinuria to determine underlying pathways of cystinuria renal damage using high throughput RNA sequencing. In all, the transcriptional sequencing data of type B cystinuria rat strain may provide invaluable resources for mechanistic and therapeutic studies.

## Materials and methods

### Microinjection of sgRNA and Cas9 mRNA

The CRISPR-Cas9 editing protocol was performed according to Yanjiao et al. [22]. Zygotes obtained from super-ovulated pregnant female Sprague Dawley rats (SLAC, Shanghai) were cultured in KSOM embryo culture medium (Millipore, USA) for 3–4 h at 37 °C, 5% CO_2_ before gene editing. Editing was performed by microinjection of TE solution containing 12.5 ng/μL of sgRNA-targeting exon 3 of the rat *Slc7a9* gene, and 25 ng/μL of Cas9 mRNA into the embryos’ cytoplasm during the one-cell stage. After injection, those zygotes were immediately transferred into pseudopregnant female rats.

### Gene sequencing

We extracted genomic DNA of single clones from tail tips and the target gene was amplified by PCR, using allele-specific primers (Supplementary Table S2). PCR was performed using EasyTaq DNA Polymerase for 30 cycles of 10 s at 98 ℃, 30 s at 55 ℃ and 1 min at 72 ℃. The PCR products were validated using 2% agarose gels. All genomic DNA was sent to Sangon Biotech (Shanghai, China) for DNA Sanger sequencing.

### Development and breeding of *Slc7a9*-deficient rats

Heterozygous *Slc7a9* rats (#2 and #4) and a homozygous *Slc7a9*-deficient rat (#8) were intercrossed to produce homozygous *Slc7a9*-deficient rats. Homozygous *Slc7a9*-deficient and WT rats as controls were used to obtain phenotypic data. Rats were bred and maintained in a pathogen-free facility, with free access to standard irradiated chow and autoclaved water. Environmental enrichment was provided to all animals, and every experiment utilizing rats was performed in compliance with guidelines established by the Animal Welfare Act for housing and care of laboratory animals, and was conducted with the approval of the Xinhua Hospital Animal Care and Use Committee.

### Off-target site analysis

All off-target sites for each sgRNA were predicted and we selected the top ten candidates using a published online prediction tool [23] (http://www.rgenome.net/cas-offinder/). Table S1 provides a list of ten target sites. The analysis was performed via PCR of 400 bp fragments flanking the off-target cut sites, and the PCR products were sequenced directly.

### Real-time quantitative PCR

Total RNA was extracted from the kidney of *Slc7a9*-deficient rats and WT rats using TRIzol reagent (Invitrogen) and cleaned further using a RNeasy kit (Qiagen). cDNA synthesis was performed with HiScript® III RT SuperMix for qPCR (Vazyme, Nanjing, China) with random primers to achieve reverse transcription of total RNA. The expression of *Slc7a9* mRNA was determined using a QuantStudio Q3 system (ABI), with a SYBR kit (Yeasen, China) in a solution system of 20 μl including 2 μl of cDNA, 0.4 μl primers each and 10 μl of SYBR-Green mix. The relative mRNA expression levels were calculated using the comparative cycle threshold (Ct) method normalized to the housekeeping gene GAPDH at the mRNA level. The primers of gene *Slc7a9* and β-actin are listed in Supplementary Table S2. Four replicates were used both for each targeted gene and for the test strains [24].

### Western blotting

Total protein was extracted from the frozen kidney tissue using 8 M urea lysis buffer (Sigma, U5128) containing PMAF (Beyotime, China). Lysates were harvested for protein analysis for 30 min on ice, and centrifuged at 20,000 *g* for 15 min, with the supernatant used for western blotting. Protein concentration was determined using BCA assays (Yeasen, China). Equal amounts of protein concentration were diluted in 5 × SDS loading buffer (Yeasen, China) and heated to 99 °C for 5 min and separated using 10% SDS-PAGE. Proteins were transferred onto PVDF membranes (Millipore, USA) and blots were blocked with 5% bovine serum albumin dissolved in Tris-buffered saline (TBS) containing 0.1% Tween-20 (TBST) at room temperature for 1 h, and incubated with primary antibodies overnight at 4 °C. After washing the blots three times with TBST, the membranes were incubated with an appropriate HRP-conjugated secondary antibody (Cell Signaling Technology, USA) for 1 h at room temperature, and washed again with TBST. Protein immunoblots were finally visualized using electrogenerated chemiluminescence (ECL, Pierce Biotechnology, USA) with the Bio-Rad ChemiDoc XRS imaging system. Primary antibody to *Slc7a9* (Cat. #ab203385) and β-actin (Cat. #ab8227) was purchased from Abcam, UK. The blots were quantitatively analyzed using Image J software (X64, v. 2.1.4).

### Urinary determination

We collected urine from 20 rats (*Slc7a9*-deficient and WT rats, ten rats in each group, half male and half female), and every sample was measured in the same measurement three times. Urine samples were collected in metabolic cages without food provided for 24 h. Liquid chromatography and tandem mass spectrometry (LC–MS/MS) were performed for urinary cystine determination. Standard of cystine was obtained from Sigma-Aldrich (St. Louis, MO, USA). N152-cystine was from Cambridge Isotope Laboratories, Inc. (Andover, MA, USA). Acetonitrile and methanol were from Merck (Darmstadt, Germany) of the highest purity grades. LC–MS/MS was performed on an Acquity UPLC device connected to a Xevo TQ-S mass spectrometer (Waters, MA, USA). The column used was a Waters Acquity HSS T3 column (1.7 μm, 100 × 2.1 mm) maintained at 40 °C. Mobile phases consisted of 0.1% formic acid in water and 0.1% formic acid in Acetonitrile. Total run-time was 7 min and the injection volume was 2 μL. All samples were measured in a positive electrospray ion mode.

The same urine sample was used for urinary creatinine determination by creatinine kit, sarcosine oxidase-PAP method (KHB, Shanghai) and was measured by KHB 310 (KHB, Shanghai), an automatic biochemistry analyzer.

To search for urinary crystals, fresh urine was collected in a metabolic cage and centrifuged at 5000 ×*g* for 15 min and the sediments were resuspended in 1/20th of the original volume. The suspension was observed directly under a microscope (magnification, × 20).

### Assessment of kidney injury

Each kidney tissue was fixed in 4% buffered paraformaldehyde and embedded in paraffin immediately after excision. After being dehydrated, cleared and embedded, paraffin-embedded samples were cut into 3-μm sections for histopathological examination. Tissue sections were de-paraffinized in xylene and stained with HE for morphological evaluation. Masson’s trichrome was used to reveal fibrosis. For immunohistochemistry, kidney slides were stained with anti-*Slc7a9* antibody (Abcam, UK). Renal cortical and medullary cell death was determined respectively, using terminal deoxynucleotidyl transferase dUTP nick-end labeling (TUNEL) staining using a specific kit (yeasen, Shanghai). Histopathological examinations of the kidneys were performed by an independent pathologist and one of the investigators.

### RNA-sequencing and bioinformatic analysis

RNA was isolated using the TRIzol reagent (Invitrogen Life Technologies). Three micrograms of RNA were used as input material for the RNA sample preparations, and sequencing libraries were generated using the TruSeq RNA Sample Preparation Kit (Illumina, San Diego, CA, USA). To prepare for hybridization, Illumina PE adapter oligonucleotides were ligated after adenylation of the 3′ ends of the DNA fragments. Selected cDNA fragments of the preferred 200 bp in length, with ligated adaptor molecules on both ends, were selectively enriched using Illumina PCR Primer Cocktail in a 15 cycle PCR reaction. After purifying (AMPure XP system) and quantifying products using the Agilent high sensitivity DNA assay on a Bioanalyzer 2100 system (Agilent), the sequencing library was then sequenced on a HiSeq platform (Illumina) by Shanghai Personal Biotechnology Cp. Ltd. DEGs were analyzed using various bioinformatics methods to reveal the mechanism of cystinuria. RNA expression levels were measured as total exon reads/mapped reads (millions) x exon length (kb). DEGs were evaluated at log2 fold‑change (FC) threshold (|FC|> 1.5). The annotation and functional enrichment of differentially expressed genes were performed, and GO and KEGG databases provided arranged genes of specific informative groups, in Gene Ontology (GO; http://www.geneontology.org/) and Kyoto Encyclopedia of Genes and Genomes (KEGG; www.genome.jp/kegg/). GO functional items of DEGs were assigned to three categories (biological process, cellular component, and molecular function). *P* < 0.05 was considered to indicate a statistically significant difference.

### Statistical analysis

Every sample was technically repeated at least three times, and values are mean ± SD. Student’s *t*-test was used to evaluate the significance of group differences. *P*-values less than 0.05 were considered significant. Statistical data were calculated using SPSS 11.5 software (SPSS, Inc., Chicago, IL, USA).

## Results

### Targeted deletion of the *Slc7a9* gene using the CRISPR-Cas9 system in rats

**Fig. 1:**
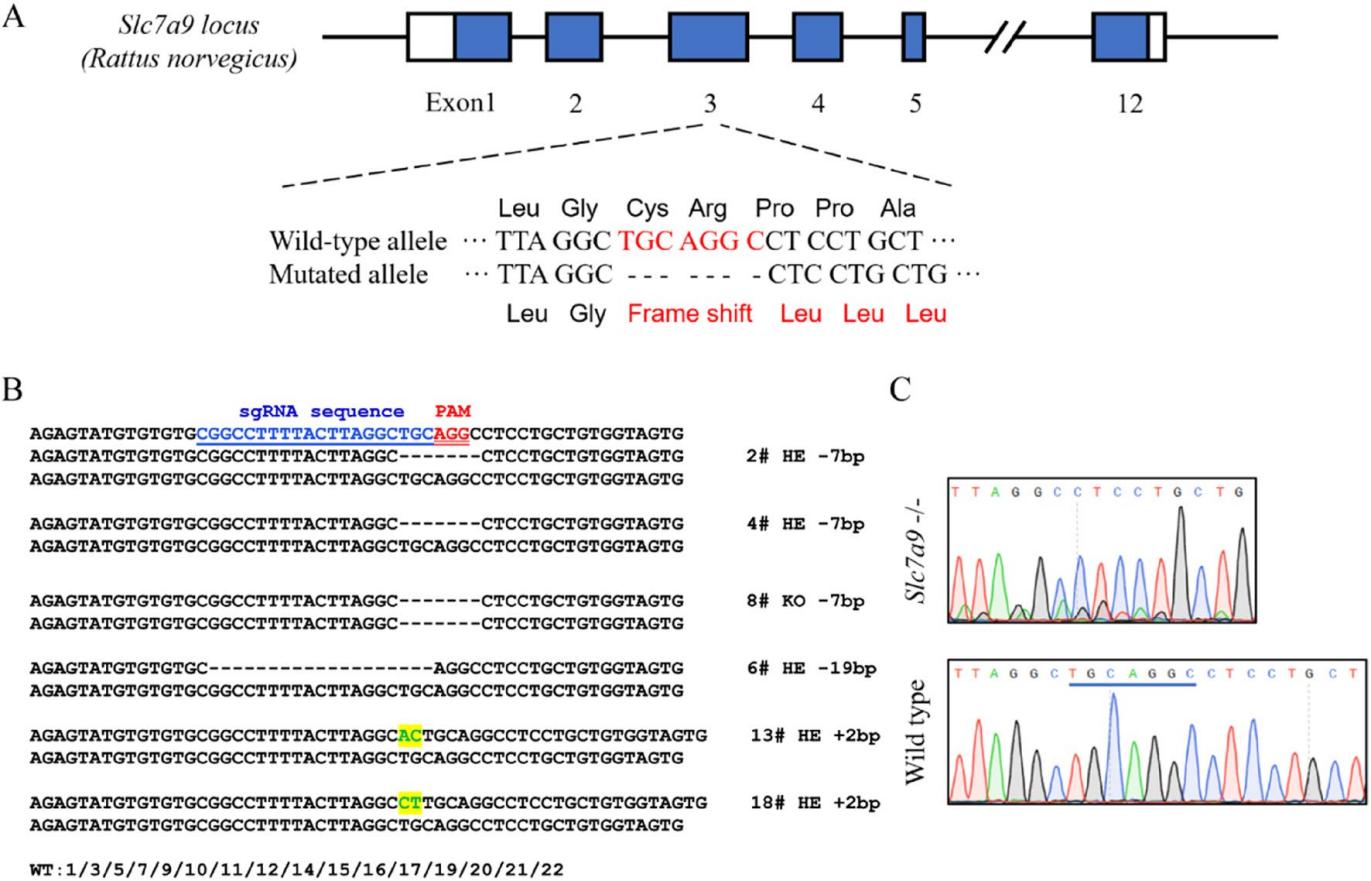
Generation of heritable *Slc7a9*-deficient rats using the CRISPR-Cas9 system. **A** Schematic diagram of *Slc7a9*-deficient rat model construction using CRISPR-Cas9. The targeted sgRNA sequence at exon 3 is aligned in a single solid line and the PAM sequence is aligned using a double line. **B** Discriminations of *Slc7a9* gene DNA sequence in recombinant clones. SgRNA in blue is labeled by a single line and the PAM in red is indicated by a double line. The dashed line and highlight stand for deletion and insertion of nucleotides, respectively. **C** Sequencing result of homozygous *Slc7a9*-deficient and wild-type rats. The missing 7 base pair (bp) sequence is indicated

We knocked out 7 bp in exon 3 of the *Slc7a9* gene using the CRISPR-Cas9 system (Fig. 1A). This resulted in a frameshift mutation that created an early-stop in transcription. Tail tips for PCR amplification and TA-cloning followed by Sanger sequencing were cut to extract genomic DNA from the pups. Six of 22 pups were identified as founders with different mutations in the *Slc7a9* gene (Fig. 1B). Among these, #2 and #4 were heterozygotes and #8 was a homozygote. After intercrossing founders #2, #4, and #8, we confirmed that the exact *Slc7a9* mutation in the F1 rats represented a heritable strain on the Sprague Dawley genetic background with the *Slc7a9* gene knocked out (Fig. 1C). The CRISPR-Cas9 system has the potential for creating off-target effects because the selected target sequence of CRISPR-Cas9 allows a 1–3 base pair matching error [25–28]. We examined ten predicted off-target sites (Supplementary Table S1) using deep sequencing of founder genomic DNA and found no off-target cleavages. All verifications and experiments mentioned later were based on the homozygous rats.

### Lacking of *Slc7a9* expression in *Slc7a9*-deficient rats

**Fig. 2:**
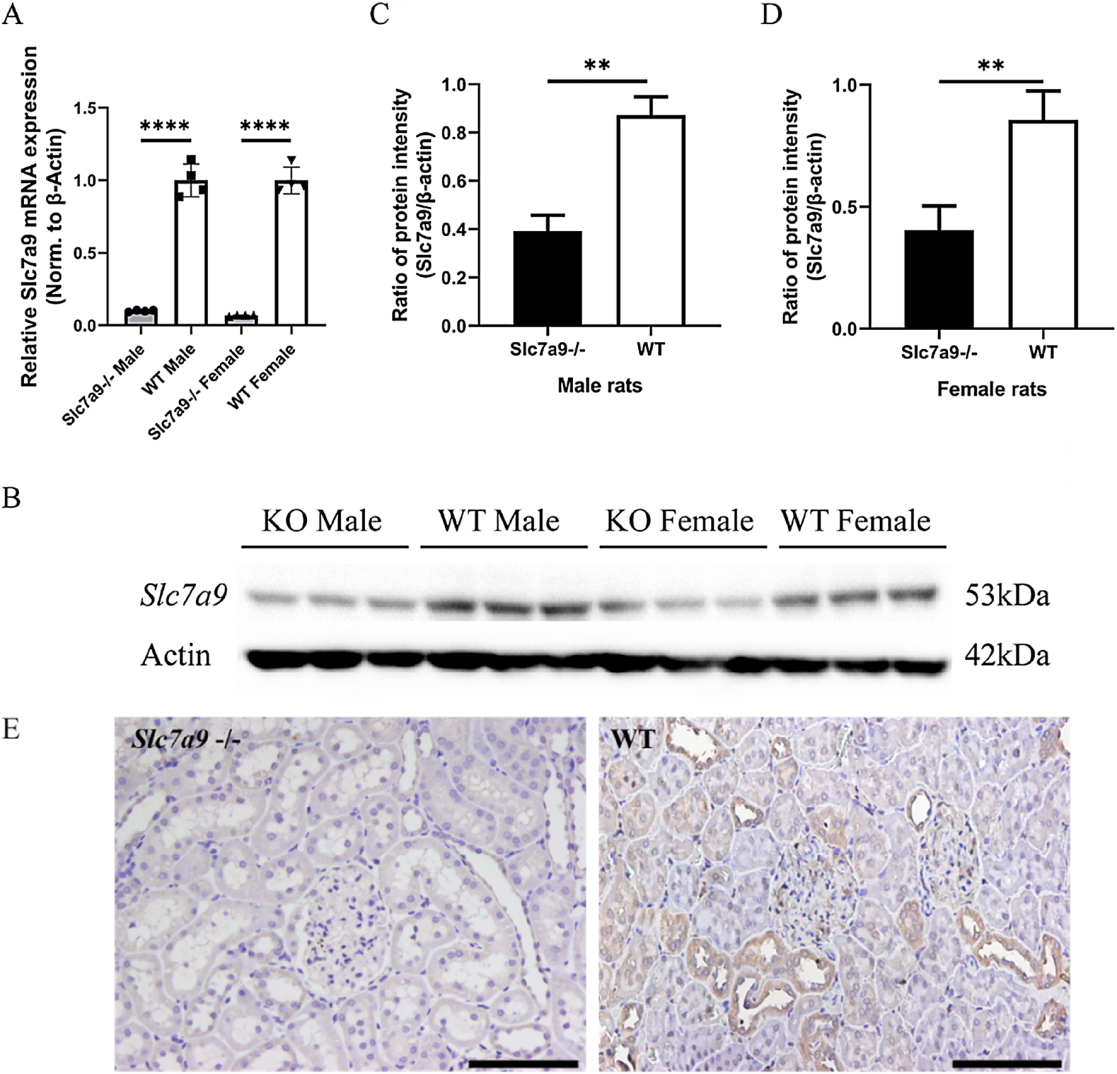
mRNA and protein expression of cystine transporter *Slc7a9*/b^0,+^AT in rat kidney. **A** Quantitative PCR analysis of rat kidney RNA from different gender, WT (*n* = 4) and *Slc7a9*-deficient rats (*n* = 4), ****P < 0.0001, *t*-test. **B–D** Western blot and quantification of protein b^0,+^AT, encoded by *Slc7a9* in WT and *Slc7a9*-deficient rats. Cropped blots were presented. The samples were derived from the same experiment and gel/blot were processed in parallel. b^0,+^AT is revealed as one protein band of 53 kD, β-actin is used as a loading control, and appears as one 42kD protein band, **P < 0.01, *t*-test. *E* Immunohistochemistry staining of *Slc7a9*-deficient rat (Left of Fig. 2E) and SD rat (Right of Fig. 2E.b) using *Slc7a9* antibody. Black scale bar = 100 μm

At the level of gene transcription, quantitative PCR demonstrated significantly lower *Slc7a9* expression in the kidneys of homozygous mutants compared to WT rats of both sexes (Fig. 2A). With respect to translation, regardless of sex, western blot analysis demonstrated a distinct visual reduction of b^0,+^AT in the kidney tissue of *Slc7a9*-deficient rats in comparison to WT rats (Fig. 2B–D). Although the expression amount of b^0,+^AT was apparently reduced according to the statistical results, the band that indicated b^0,+^AT could still be seen in the kidney tissue protein of *Slc7a9*-deficient rats using western blot. This might have been caused by antibody binding with the incomplete protein generated by early-terminated translation of frame-shift mutation. To validate biochemical phenotypes at the tissue level, sections of renal cortex tissue were subjected to immunochemistry (IHC) to study in situ expression of b^0,+^AT in *Slc7a9*-deficient rats (Fig. 2E). Consistent with the western blot result, the immunochemistry results showed b^0,+^AT enriched in the proximal renal tubules in WT rats, and relatively low expression levels in *Slc7a9*-deficient rats.

### Urinary phenotype of *Slc7a9*-deficient rats characterized cystinuria

**Fig. 3:**
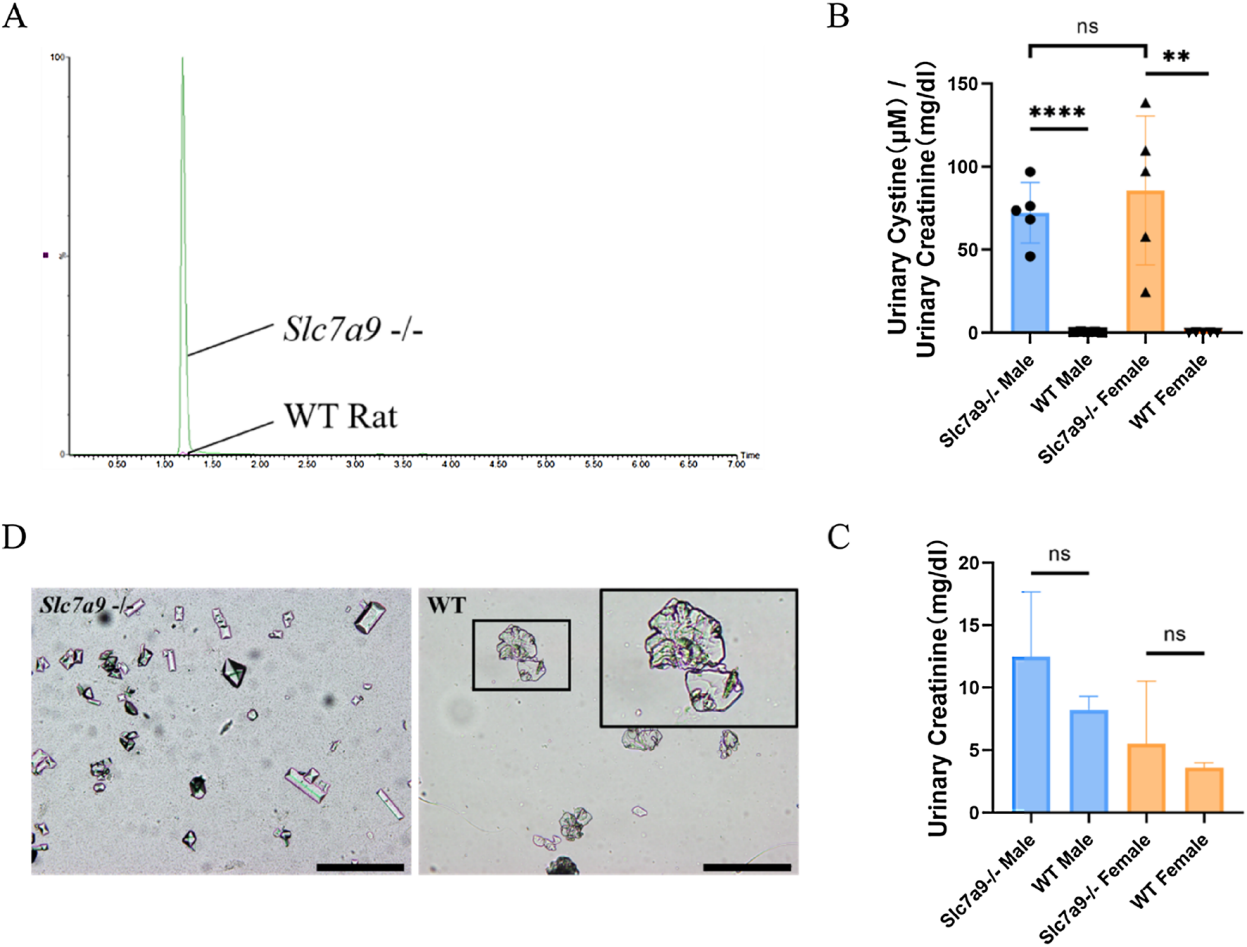
Urinary cystine level of *Slc7a9*-deficient and WT rats. **A** Comparison of the chromatograms of urinary cystine between *Slc7a9*-deficient and WT rats. The retention time for cystine in analyte is identical at 1.18 min. **B** Relative urinary cystine concentration for WT rats of male and female (each sex, *n* = 5), *Slc7a9*-deficient rats of male and female (each sex, *n* = 5). Cystine in urine was measured using liquid chromatography-tandem mass spectrometry. Values are normalized to creatinine measurements. Data are presented as means ± standard error. Significant differences in urinary concentration were determined using Student’s *t*-test. **C** Urinary creatinine concentration for WT rats of male and female (each sex, *n* = 5), *Slc7a9*-deficient rats of male and female (each sex, *n* = 5). There is no significant difference between the *Slc7a9*-deficient and WT groups in male and female rats. **D** Typical flat hexagonal cystine crystals were observed in urine sediments of 6-month-old *Slc7a9*-deficient rats that were absent in the sediments of WT rats. The inset is the local magnification. Black scale bar = 100 μm

The symbolic phenotypic traits of cystinuria in humans are urinary hyperexcretion of cystine and other dibasic amino acids including ornithine, lysine and arginine (replaced with COLA in the later text), and calculi in some patients. Type B cystinuria is attributed to mutations in SLC7A9 [2]. Based on clinical presentations, we recorded 24-h urinary cystine excretion by LC–MS/MS (Fig. 3A–C) and searched for urinary crystals (Fig. 3D) to validate biological phenotypes of *Slc7a9*-deficient model rats. To avoid sex impacts on the severity of type B cystinuria [9], we collected 24-h urine from 2-month-old *Slc7a9*-deficient and WT rats of both genders. Urinary creatinine concentrations (Fig. 3B) were measured to standardize relative urinary cystine excretion so as to eliminate the impact of basal metabolism. Relative cystine excretion was such that male *Slc7a9*-deficient rats showed significant hyperexcretion of cystine compared with WT rats with barely any excretion of cystine (Fig. 3A, B); this tendency was also seen in the female rats and the difference between sexes showed no significance. Three other dibasic amino acids (arginine, ornithine, and lysine) were also found hyperexcreting in *Slc7a9* −/− rat model (Supplementary Figure S1). Urine sediments were examined under a microscope to identify crystalluria. Similar to human classic cystinuria, cystine hexagonal crystals were observed in the urine of *Slc7a9*-deficient rats, instead of common crystals (calcium oxalate crystal, calcium phosphate crystal and ammoniomagnesium phosphate crystal) in the urine of WT rats (Fig. 3D). Interestingly, though the *Slc7a9*-deficient rats showed features of cystinuria such as hyperexcretion of cystine and cystine crystalluria, stones were not found in any model rat at any age after examining the entire urinary system (bladder, ureters and renal pelvis).

### Renal medullary fibrosis as evidence of renal injury in *Slc7a9*-deficient rats

**Fig. 4:**
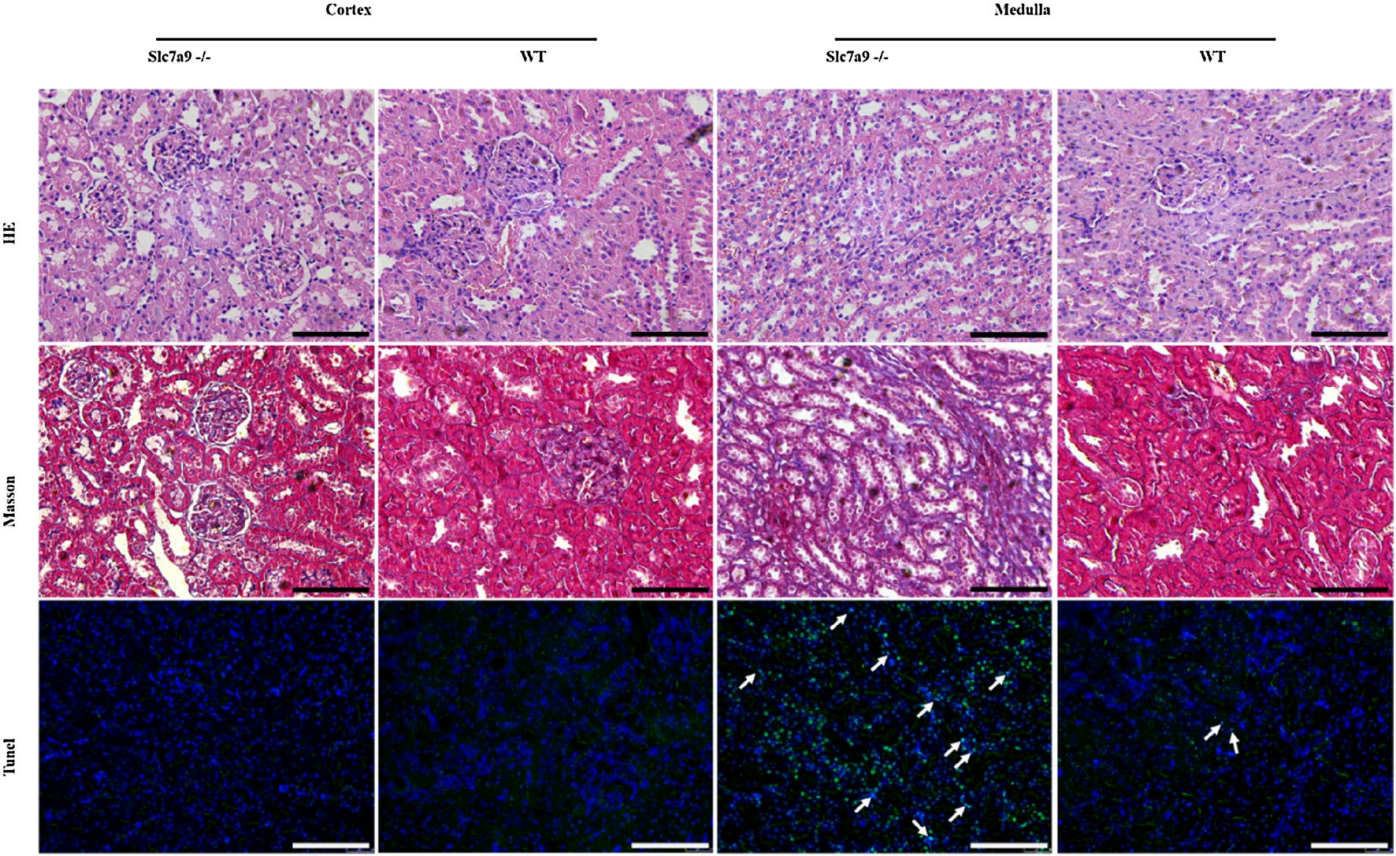
Evidence of renal injury in *Slc7a9*-deficient rats. Black and white scale bar = 100 μm. Renal sections stained by Hematoxylin & eosin (HE) staining, Masson’s trichrome staining and TUNEL staining, in comparison of kidney injury level in cortex and medulla of *Slc7a9−*/*−* and WT rats. Section of *Slc7a9*-deficient rat presented mild tubulointerstitial fibrosis (stained in blue). In TUNEL staining, positive cell (green), DAPI (blue), and white arrows pointing the TUNEL and DAPI labeled nuclei. In all pictures, scale bar = 100 μm

Renal histopathology indicated typical secondary changes of kidney damage (Fig. 4). To observe renal damage caused by cystinuria and evaluate the pathophysiological changes in the kidney in the model rat, sections of cortex and medulla of model rats and WT rats was stained in different methods. In kidney sections of *Slc7a9*-deficient rat, though hematoxylin/eosin (HE) stained sections showed barely cystine crystal deposition and dilation of tubules and glomeruli, Masson staining (collagenous fiber was stained in blue) and TUNEL test (arrows point to positive cells) suggested that model rat had more sever degree fibrosis and apoptosis phenomenon in medulla than WT rat. The results presented that renal damage of *Slc7a9*-deficient rat behaves as increasing tubulointerstitial fibrosis in renal glomerular tissue even without stone formation in the urinary track.

### Expression profiling of *Slc7a9*-deficient rats by RNA Seq uncovered metabolic transformations in cystinuria

**Fig. 5:**
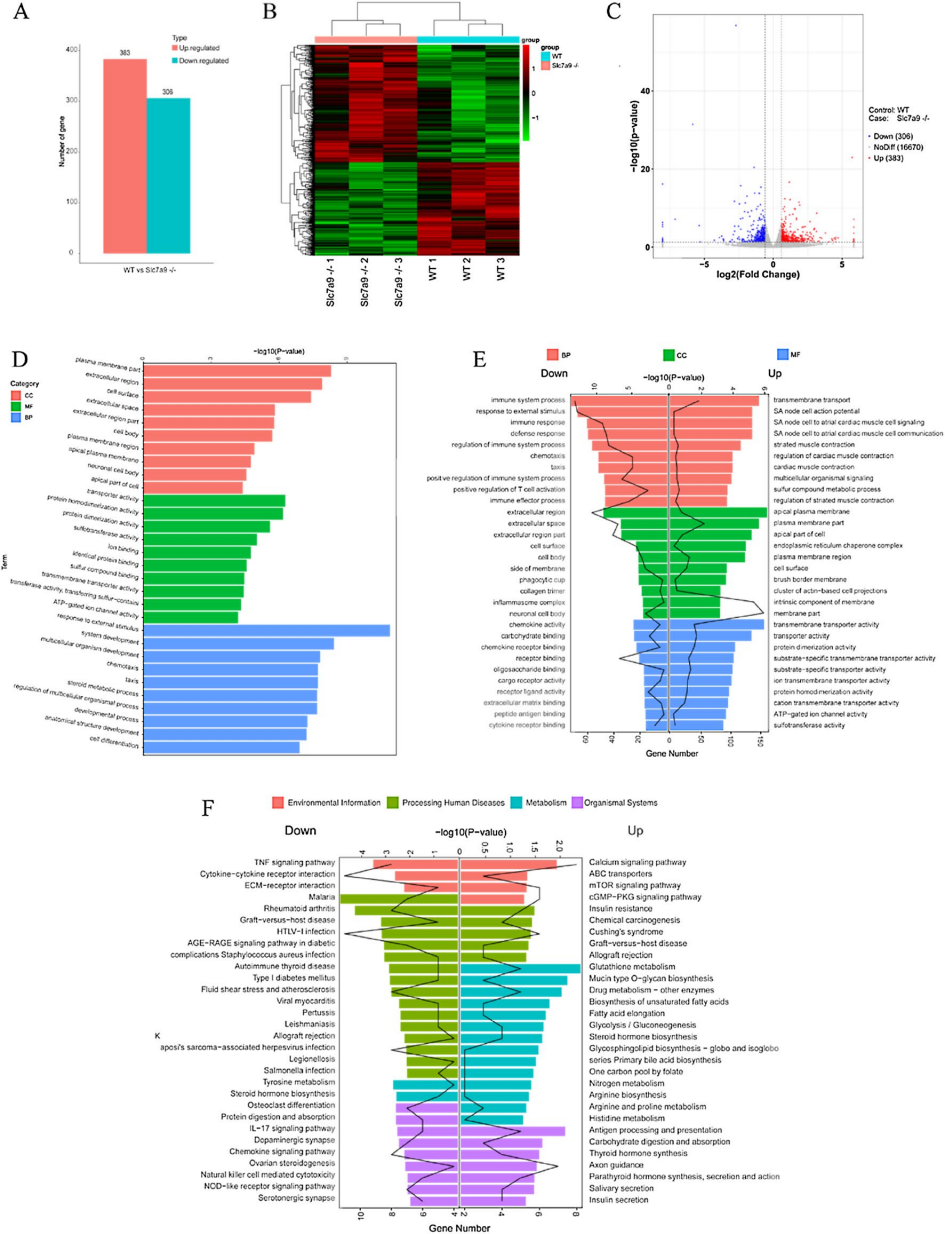
RNA-Sequencing (RNA-Seq) analysis of three 6-month-old female *Slc7a9*-deficient model rats and three WT control rats of the same age, as well as verification. **A** Statistic of DEGs. 689 are significantly differentially expressed in *Slc7a9*-deficient model rat (FC = 1.5), 383 of which are up-regulated (red dots) and 306 are down-regulated (blue dots). **B** Cluster dendrogram and heatmap of differentially expressed mRNA. **C** Volcano plot of RNA-Seq data. Up-regulated (red dots) and 306 are down-regulated (blue dots). **D** Gene Ontology enrichment analysis of DEGs. Each classification presents the top 10 GO term by FDR value. **E** Gene Ontology (GO) enrichment analysis of genes that are differentially expressed in *Slc7a9*-deficient model rat. Down-regulated GO terms are listed at the left and up-regulated are listed at the right. Bars indicated for -log10 (p-value), polyline indicated for numbers of genes. **F** Pathways analysis by KEGG database. The top 30 most represented pathways of up- and downregulated pathways in the cortical kidney. Bars indicated for -log10 (*p*-value), polyline indicated for numbers of genes

To understand the overall impact of deleting *Slc7a9* more comprehensively, as well as the impact of cystinuria, kidney cortex tissues of three female *Slc7a9*-deficient rats and three WT female rats were used for high-throughput RNA sequencing. Differentially expressed transcripts were evaluated at log2 fold-change thresholds (|FC|> 1.5). Altogether, 689 differentially expressed gene (DEGs) was revealed with 383 genes upregulating and 306 genes downregulating (Fig. 5A). A visual representation of DEGs of model and WT rats is shown as heatmap and volcano image (Fig. 5B, C). The most significant DEGs is Slc7a9, which verified the accuracy of sequencing data. To perform an unbiased annotation of the functions of the DEGs, GO functional enrichment analysis was carried out. The top ten significantly differentially gene annotation terms of CC, MF and BP in *Slc7a9*-deficient rats (in control of WT rats) pointed out those obviously changed biological function (Fig. 5D). GO analysis generated a list of up- and downregulation of genes (Fig. 5E). Expression of response to external stimulus, plasma membrane part, extracellular region, steroid metabolic process, cell surface, and other terms regarding transmembrane transport distinguished between WT and *Slc7a9*-deficient groups. Among the first three upregulated groups in terms of p-value, apical plasma membrane included 17 relevant genes, followed by 38 genes grouped under transmembrane transporter activity and 46 genes grouped under transmembrane transport. To identify the biological pathways that were activated or inhibited after the deletion of *Slc7a9*, KEGG pathway enrichment analysis was performed (Fig. 5F). DEGs were mapped to 60 statistically significant pathways (*p* < 0.05; Table 1). Mapping of common differential transcripts to the KEGG cancer signaling pathway noted that glutathione metabolism, the mTOR signaling pathway, protein digestion and absorption, and other metabolic-related pathways changed significantly. Among these pathways, the glutathione metabolism pathway showed the most increased expression levels in metabolism processes of *Slc7a9*-deficient rats, while expression levels of the TNF signaling pathway were reduced. These findings suggest for the first time that the genetic characteristic of cystinuria that deficiency in *Slc7a9* could results in the transformation of other biological processes, especially in the function of biological membrane; these findings also hint that, in addition to genetics, there are other regulatory pathways that related to the development of cystinuria which deserve further exploration. The raw sequencing reads can be obtained in the Gene Expression Omnibus (GEO) database with accession number GSE178871.

**Table 1:** Total 60 statistically significant pathways of KEGG analysis, ranked by p-value

Pathway ID	Pathway	DEG_number	Pvalue
rno00140	Steroid hormone biosynthesis	9	0.000187
rno05320	Autoimmune thyroid disease	8	0.000322
rno04940	Type I diabetes mellitus	8	0.000368
rno05323	Rheumatoid arthritis	10	0.000482
rno00480	Glutathione metabolism	8	0.00076
rno05330	Allograft rejection	7	0.000826
rno05144	Malaria	7	0.001358
rno05416	Viral myocarditis	8	0.001437
rno04933	AGE-RAGE signaling pathway in diabetic complications	10	0.001474
rno04612	Antigen processing and presentation	8	0.002306
rno00910	Nitrogen metabolism	4	0.002498
rno05418	Fluid shear stress and atherosclerosis	12	0.002649
rno05166	HTLV-I infection	16	0.002684
rno04728	Dopaminergic synapse	11	0.003059
rno04668	TNF signaling pathway	10	0.003256
rno04650	Natural killer cell mediated cytotoxicity	10	0.003961
rno00830	Retinol metabolism	7	0.004228
rno05150	Staphylococcus aureus infection	6	0.004789
rno04614	Renin-angiotensin system	5	0.004877
rno04145	Phagosome	12	0.005118
rno00350	Tyrosine metabolism	5	0.005539
rno04380	Osteoclast differentiation	10	0.006076
rno05167	Kaposi's sarcoma-associated herpesvirus infection	13	0.006567
rno04514	Cell adhesion molecules (CAMs)	11	0.008318
rno04974	Protein digestion and absorption	8	0.009166
rno00982	Drug metabolism—cytochrome P450	6	0.009328
rno04926	Relaxin signaling pathway	10	0.009984
rno04713	Circadian entrainment	8	0.010422
rno04657	IL-17 signaling pathway	8	0.010422
rno00983	Drug metabolism—other enzymes	7	0.011048
rno00120	Primary bile acid biosynthesis	3	0.014602
rno05031	Amphetamine addiction	6	0.015153
rno05164	Influenza A	11	0.015918
rno05168	Herpes simplex infection	12	0.017247
rno01040	Biosynthesis of unsaturated fatty acids	4	0.018327
rno04625	C-type lectin receptor signaling pathway	8	0.01868
rno04918	Thyroid hormone synthesis	6	0.020207
rno04913	Ovarian steroidogenesis	5	0.020619
rno04151	PI3K-Akt signaling pathway	18	0.021211
rno04621	NOD-like receptor signaling pathway	10	0.024182
rno05134	Legionellosis	5	0.024191
rno05133	Pertussis	6	0.024674
rno05140	Leishmaniasis	6	0.024674
rno04976	Bile secretion	6	0.024674
rno04060	Cytokine-cytokine receptor interaction	15	0.024789
rno04979	Cholesterol metabolism	5	0.026117
rno00360	Phenylalanine metabolism	3	0.026969
rno04973	Carbohydrate digestion and absorption	4	0.027518
rno05146	Amoebiasis	7	0.02787
rno04970	Salivary secretion	6	0.027991
rno05204	Chemical carcinogenesis	6	0.031591
rno00340	Histidine metabolism	3	0.03471
rno04512	ECM-receptor interaction	6	0.03548
rno00980	Metabolism of xenobiotics by cytochrome P450	5	0.037201
rno00512	Mucin type O-glycan biosynthesis	3	0.038965
rno04610	Complement and coagulation cascades	6	0.039664
rno04927	Cortisol synthesis and secretion	5	0.045048
rno05132	Salmonella infection	6	0.046505
rno04934	Cushing’s syndrome	9	0.047935
rno00500	Starch and sucrose metabolism	3	0.048227

## Discussion

Animal models of human diseases provide in-depth knowledge of pathophysiology and guide the development of treatments using innovative treatment protocols. We generated and characterized the first rat model of type B cystinuria using the CRISPR-Cas9 system. This model matches genetic subtype of type B cystinuria [29, 30] both in terms of genetics (defect in *Slc7a9* gene) and phenotype. The phenotype of *Slc7a9*-deficient rats (hyperexcretion of urine cystine, crystalluria and mild inflammatory renal pathology changes) resembles that of clinical cystinuria. On the basis of this disease model, we searched for new pathogenic mechanisms of cystinuria.

Analysis of the genome of model rats verified the absence of *Slc7a9* gene, encoding the amino acid transport system b^0,+^AT, which plays a major role in cystine reabsorption in the kidney [31]. With frameshift alleles, relative quantities of renal b^0,+^AT in *Slc7a9*-deficient rats were reduced by over 50% compared with WT rats., suggesting the disruption of *Slc7a9* gene. These findings together suggest that our CRISPR-Cas9 knockout method led to reduced b^0,+^AT expression in terms of both transcription and translation.

One of the most striking findings observed in the *Slc7a9*-deficient rats was marked hyperexcretion of urinary cystine compared with WT rats. In addition, typical hexagonal cystine crystals can be observed in both cystinuria patients and our *Slc7a9*-deficient rat models. A previous study of a cystinuria mouse model reported that most of their *Slc7a9* −/− mice developed cystine stones, but still seven mice appeared non-lithiasic [20]. In our study, no cystine stones were found in the urinary system of *Slc7a9*-deficient rats, though cystine crystals can be induced by reducing water intake in *Slc7a9*-deficient rats. Although cystine supersaturation is a key factor, the pathogenesis of stone development is complex [31]. Accordingly, not all patients with cystinuria develop urinary stone throughout their lives [32]. This suggests to us those other mechanisms are involved in the process of stone formation. Interestingly, as a non-exclusive union of rBAT protein, a second cystine transporter partner of rBAT compensating similar function of b^0,+^AT was found in the apical membrane [33, 34]. These results suggest that *Slc7a9*-deficient rats simulate clinical cystinuria in terms of biochemical phenotype. Therefore, further studies of lithiasis-modulating genes and conditions using animal disease model are indispensable for exploring stone formation in cystinuria, making this rat model valuable.

Another important observation is that *Slc7a9*-deficient rats exhibited moderately renal injury seen on pathology. Over 70% of patients with cystinuria may suffer from CKD [12, 13], characterized by inflammatory and fibrotic changes of surrounding interstitium caused by plugging with cystine crystals. In the long term, repetitive inflammation and fibrillation lead to nephron dysfunction and CKD [35]. In type B cystinuria mouse model, kidney damage was also found in mice without calculi formation [20]. As many as 5.8% of patients with cystinuria demonstrated increased serum creatinine concentrations [36], and 27% had reduced estimated glomerular filtration rates. In the present study, mild kidney abnormalities of *Slc7a9*-deficient rats included slight dilatation of renal tubules in the cortical area and increased collagenous deposition around the kidney, which exactly corresponds to the evidence to the effect that cystinuria leads to chronic nephritis. These pathological changes observed in *Slc7a9*-deficient rats support the notion that the model rats correlated well in terms of pathophysiological processes.

Further, we performed high throughput RNA sequencing using our *Slc7a9*-deficient rats. We found that, after *Slc7a9* was artificially reduced in rats, terms for membrane and transmembrane transport showed significant changes, and KEGG enrichment analysis indicated that pathways of GSH metabolism might function during cystinuria. GSH, a tripeptide consisting of the amino acids glutamate, cystine and glycine, is almost ubiquitous in biological systems [37]. Mainly acting as an endogenous antioxidant, GSH plays an important role in the detoxification of xenobiotics and their metabolites, maintaining the intracellular redox balance [38, 39]. It is reported that decreased GSH concentration in cellular is an early event in the apoptotic cascade induced by death receptor activation [40], mitochondrial apoptotic signaling [41], and oxidative stress [42]. While in some situations the rates of GSH production cannot maintain normal cellular concentrations of GSH during certain disease or stressful state, then oxidative stress level increased and damages happen. Cystine is the rate-limiting amino acid in the synthesis of GSH [43], and lipoic acid (LA), which can prevent cystine urolithiasis in a cystinuria mouse model, has been shown to eliminate toxic substances by enhancing intracellular GSH level [44]. All the data above hinted that the relevance between GSH pathway participating mechanism of cystinuria, though there has been no research reported yet. The activated GSH pathway in the kidney of cystinuria rat may explain the renal damage of *Slc7a9*-deficient rat and provide some clues regarding pathogenic and therapeutic directions. Changes in membrane function, activity, and stability in GO functional analysis of *Slc7a9*-deficient rats predicted that defective *Slc7a9* affected not only the transport function of b^0,+^AT, but also many other aspects of membrane and unknown functions. These findings may lead to a better understanding of the factors modulating the severity of cystinuria, and to uncovering underlying mechanisms of this disease with respect to structure and membrane components.

## Conclusions

In summary, we generated the first *Slc7a9*-deficient model using CRISPR-Cas9 gene editing system in rats. This is the first rat model that successfully simulated type B cystinuria in terms of gene transcription, translation, biological phenotypes, and pathological changes without off-target mutations. We created the first cystinuria transcriptomic database and proposed the hypothesis of a connection between renal kidney injury of homocystinuria and GSH metabolism. This *Slc7a9*-deficient rats represent an ideal model to clarify the pathophysiology of cystinuria and to provide a favorable animal model for further clinical studies.

## Supplementary Information

Below is the link to the electronic supplementary material.

Supplementary file1 (PDF 153 KB)

## Data Availability

The datasets generated during the current study id available in the following repository: GEO: GSE178871, https://www.ncbi.nlm.nih.gov/geo/query/acc.cgi?acc=GSE178871.
