# Supplementary material for: Differences in renal cortex transcriptional profiling of wild-type and novel type B cystinuria model rats
Source: Urolithiasis. 2022 Apr 13;50(3):279–91. doi: 10.1007/s00240-022-01321-6 (PMC9110498; doi:10.1007/s00240-022-01321-6)
Supplement: Supplementary file 1 — Supplementary file1 (PDF 153 KB) [file 240_2022_1321_MOESM1_ESM.pdf]

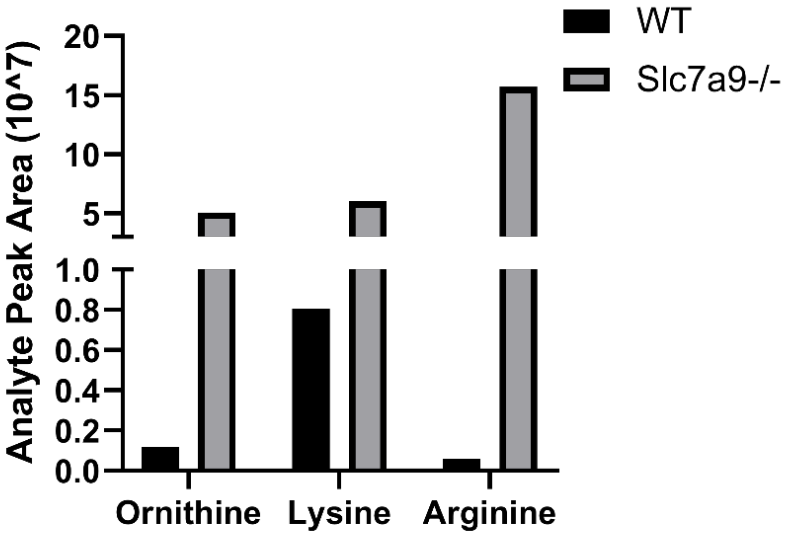

Supplementary Figure 1. Urine 24-hour dibasic amino acids analysis of *Slc7a9* -/- and WT rats by relative quantification essay.
